# Genomic Prediction for Germplasm Improvement Through Inter-Heterotic-Group Line Crossing in Maize

**DOI:** 10.3390/ijms26062662

**Published:** 2025-03-15

**Authors:** Dehe Cheng, Jinlong Li, Shuwei Guo, Yuandong Wang, Shizhong Xu, Shaojiang Chen, Wenxin Liu

**Affiliations:** 1 State Key Laboratory of Maize Bio-Breeding, National Maize Improvement Center, College of Agronomy and Biotechnology, China Agricultural University, Beijing 100193, China; 2 Maize Research Institute, Beijing Academy of Agriculture and Forestry Sciences, Beijing 100097, China; 3 Department of Botany and Plant Sciences, University of California, Riverside, CA 92521, USA

**Keywords:** genomic selection, germplasm improvement, inter-heterotic-group, maize

## Abstract

Germplasm improvement is essential for maize breeding. Currently, intra-heterotic-group crossing is the major method for germplasm improvement, while inter-heterotic-group crossing is also used in breeding but not in a systematic way. In this study, five inbred lines from four heterotic groups were used to develop a connected segregating population through inter-heterotic-group line crossing (CSPIC), which comprised 5 subpopulations with 535 doubled haploid (DH) lines and 15 related test-cross populations including 1568 hybrids. Significant genetic variation was observed in most subpopulations, with several DH populations exhibiting superior phenotypes regarding traits such as plant height (PH), ear height (EH), days to anthesis (DTA), and days to silking (DTS). Notably, 10.8% of hybrids in the population POP5/C229 surpassed the high-yielding hybrid ND678 (CK). To reduce field planting costs and quickly screen for the best inter-heterotic-group DH lines and test-cross hybrids, we assessed the accuracy of genomic selection (GS) for within- and between-population predictions in the DH populations and the test-cross populations. Within the DH or the hybrid population, the prediction accuracy varied across populations and traits, with an average hybrid yield prediction accuracy of 0.41, reaching 0.54 in POP5/Z58. In the cross DH population predictions, the prediction accuracy of the half-sib population exceeded that of the non-sib cross population prediction, with the highest accuracy observed when the non-shared parents were from the same heterotic group, and the average phenotypic prediction accuracies of POP3 predicting POP2 and POP2 predicting POP3 were 0.54 and 0.45, respectively. In the cross hybrid population predictions, the accuracy was highest when both the training and the test sets came from the same DH populations, with an average accuracy of 0.43. The proportion of shared polymorphisms with respect to SNPs between the training and the test sets (PSP) exhibited a significant and strong correlation with the prediction accuracy of cross population prediction. This study demonstrates the feasibility of creating new heterotic groups through inter-heterotic-group crossing in germplasm improvement, and some cross population prediction patterns exhibited excellent prediction accuracy.

## 1. Introduction

Maize (*Zea mays* L.) is a multi-purpose crop, serving various needs such as food, feed, economic product, and energy needs. As the global population grows and living standards increase, the demand for maize continues to rise. Breeding high-yield maize varieties has become a key solution to meet this increasing demand. Therefore, breeders must continuously improve maize germplasms. DH and GS are two important techniques for accelerating germplasm improvement. DH technology enables the rapid generation of homozygous inbred lines, shortening the breeding timeline when compared to traditional breeding methods [1]. The GS technique has been shown to enhance the rate of genetic gain in both animals and plants [2]. The accuracy of GS has been evaluated in various populations of maize [3,4,5], wheat [6,7], rice [8,9], barley [10,11], *Arabidopsis* [12], and forest trees [13,14,15]. There are many factors that affect the accuracy of GS, including the heritability of the target trait, the relationship between the training and the test populations, modeling methods, population size, the proportions in both the training and the test sets, population structure, and marker density. Highly inherited traits are phenotypically stable across multiple environments due to them being controlled by a few major genes; therefore, they have a high predictive accuracy and can achieve rapid selection responses [16,17]. The composition of the training set based on the design of the test set can help to improve the prediction accuracy [18,19,20]. Multiple models have been applied for genome prediction, including ridge regression best linear unbiased prediction (RRBLUP) [21,22], genomic best linear unbiased prediction (GBLUP) [23,24,25,26], Bayesian models [2,27,28,29,30], and machine learning models [31,32,33,34,35]. A higher marker density can increase the proportion of the genetic variation explained; however, once the number of markers reaches a certain threshold, the prediction accuracy generally reaches a plateau [16,36]. By understanding the population structure and optimizing the construction of the training set, the prediction accuracy can be improved [37,38,39]. These studies mainly focused on the evaluation of factors affecting the prediction accuracy of GS, and they have rarely evaluated the prediction accuracy of the within- and between-population prediction accuracy in inter-heterotic-group DH populations and the consequent test-cross populations.

Inter-heterotic-group-based convergent improvement is an effective method to create new heterotic groups and breed high-yield hybrids. The parents of many maize varieties are derived from the aggregation of different heterotic groups. B73 × Mo17 was the most important single-cross hybrid in the United States during the 1970s and 1980s, with Mo17 consisting of 50% Lancaster and 50% Krug (Reid) [40]. In China, utilizing foreign hybrid varieties as fundamental resources for selecting maize inbred lines is the primary approach for innovating heterotic groups. For example, the P and X groups originated from the hybrid varieties P78599 and XianYu335 [41]. Additionally, compared to other crops, maize tends to display stronger heterosis [42]. It is crucial to create new heterotic groups and establish new heterotic patterns through inter-heterotic-group crosses of DH lines [43]. To ensure the establishment of a specific heterotic pattern following inter-heterotic-group crossing, the triangular heterotic pattern serves as an effective model [44]. This is because the novel heterotic group derived from the inter-heterotic-group aggregation of any two heterotic groups exhibits a heterotic pattern in relation to the third heterotic group. However, there are fewer studies that assess inter-heterotic-group crossing germplasm improvement by genomic selection.

**Figure 1:**
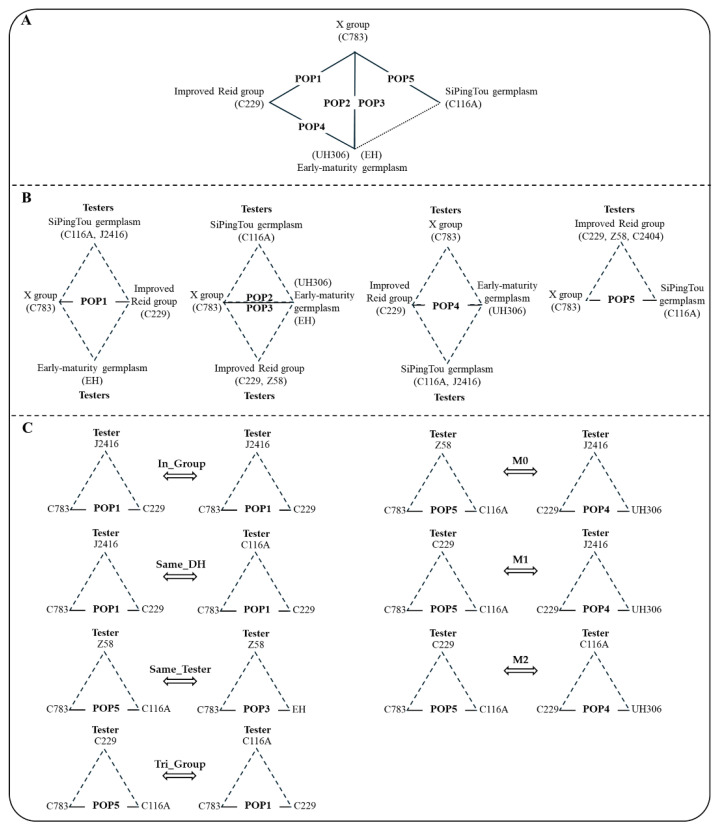
The heterotic group types of materials and the triangle pattern and prediction type of hybrids: (**A**) parental heterotic group and the connected pedigree of the core DH populations; (**B**) the triangular pattern of test-crosses for each DH hybrid population. The parents and testers belonged to three independent heterotic groups, thus forming a triangular heterotic pattern; (**C**) examples of cross hybrid population prediction types: (1) predictions within populations (In_Group); (2) predictions between different testers with the same DH populations (Same_DH); (3) predictions between different DH populations with the same testers (Same_Tester); (4) predictions within the “triangular” heterotic groups (DH parents and testers in the training and test sets sharing three identical heterotic groups, “Tri_Group”); (5–7) in addition to the above types, there were 0–2 shared inbred lines between the training and the test sets of the DH parents and testers (M0, M1, and M2, respectively).

Therefore, we developed a connected segregating population through inter-heterotic-group line crossing (CSPIC), comprising five subpopulations with 535 DH lines (Table S1, Figure 1A). To accurately evaluate the inter-heterotic-group lines, we selected three elite lines with a heterotic pattern related to the parents of the DH populations as testers and created fifteen test-cross populations, including 1568 hybrids. Among the DH population parents and the testers, a triangular relationship was settled, with the parents of the DH populations and the testers derived from three heterotic groups (Figure 1B). There are many types of population relationships in both DH and hybrid populations. Our study focused on the following key aspects related to combining DH rapid breeding technology and GS technology: (1) the genetic and phenotypic distributions of inter-heterotic-group DH lines and test-cross hybrids; (2) the elite line improvement effect through inter-heterotic-group line crossing; (3) the prediction accuracy of different types of DH cross populations (full-sib, half-sib, and unrelated); (4) the prediction accuracy of different types of hybrid cross populations (In_Group, Same_DH, Same_Tester, Tri_Group, M0, M1, M2); and (5) the influence of the genotype relationship index on the accuracy of cross population predictions for both the DH and the hybrid populations.

## 2. Results

### 2.1. Genotyping and Population Structure Analysis

To capture a broad range of genetic variation, we re-sequenced the DH populations, their parents, and the testers. A total of 108,898 SNPs were retained after quality control, and genotypes of the hybrids were synthesized through the DH lines and the testers. To investigate the genetic relationships within and between populations, principal component analysis (PCA) was performed on the DH and the hybrid populations using the filtered SNPs. The first two principal components of the DH and the hybrid populations explained 28.8% and 38.3% of the genetic variation, respectively.

**Figure 2:**
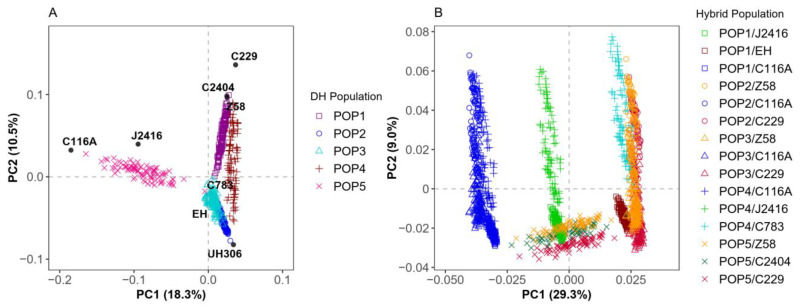
Genetic structures of the DH lines and the hybrid panels: (**A**) the principal component analysis for the DH panels; (**B**) the principal component analysis for the hybrid panels. The materials labeled in plot (**A**) represent the parents of the DH populations and the testers. In plot (**B**), the same testers share much the same colors.

In the PCA scatter plot of the DH populations, the genetic distances between the parents of the DH populations were quite large, except for the distance between C783 and EH, which belonged to different heterotic groups. In the five DH populations, only POP2 and POP3 overlapped significantly in terms of DH lines, with POP2 covering the main region of POP3. In contrast, among the other half-sibling DH populations, there was little overlap of DH lines on the PCA scatter plots (Figure 2A). The PCA scatter plot for the hybrid populations were divided into four regions. The first region included the hybrid populations of the POP1–POP4 test-crossed with C116A, which were clustered together due to the common tester C116A; the second region consisted of the hybrid populations of POP1 and POP4 crossed with J2416, which were adjacent to each other due to the common tester J2416; and the third region included POP5 test-crossed with three testers, which were clustered together due to the close genetic relationship between the three testers (i.e., C229, C2404, and Z58). The remaining hybrid populations were grouped together with their DH parents and the testers from the three divergent heterotic groups (X group, Improved Reid group, and Early-maturity germplasm) (Figure 1B and Figure 2B). There was substantial genetic variation in both the DH and the hybrid populations, with distinct patterns of aggregation observed in the hybrid populations.

### 2.2. Basic Analysis of Phenotype and Effect of Cross Heterotic Group Fusion

To investigate the impact of inter-heterotic-group fusion on trait phenotypes, we recorded DTA, DTS, PH, and EH for both the DH and hybrid populations and grain yield for the hybrid populations across multiple sites. Trait heritability and coefficients of variation were calculated for the phenotypes of the DH and their hybrid populations (Table S1). The results revealed that the phenotypic coefficients of variation were high in both the DH and hybrid populations, with the DH populations exhibiting greater variation than the test-cross populations. The coefficients of variation for yield ranged from 6.6% to 10.5% in the hybrid populations. In both the DH and the hybrid populations, the heritability of traits ranged from 0.64 to 0.96. The heritability of PH and EH was higher than that of DTA, DTS, and yield, with the heritability of yield ranging from 0.66 to 0.82. Upon observing the phenotypic distribution for each DH and hybrid population, we noted that DTA and DTS for POP1–4 were earlier than those for POP5 (Figure S1A,B). For PH and EH, the overall distribution of POP4 was low, consistent with the performance of the population founders (Figure S1C,D). All traits in the DH populations displayed super-parental inheritance. In particular, the DTS for POP5 showed a coefficient of variation of 6.3%. Although the phenotype of C116A was identical to that of C783, the progeny population exhibited substantial phenotypic variation, indicating that inter-heterotic-group fusion broadened the genetic variation (Figure S1).

**Figure 3:**
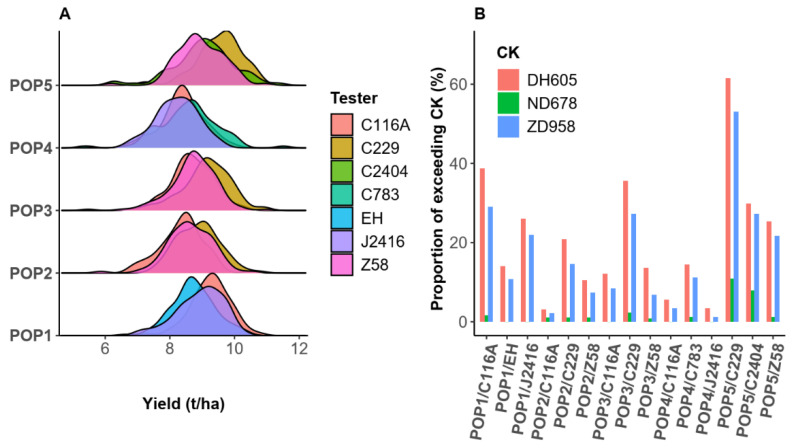
Yield distribution and proportion of hybrids exceeding the check in the hybrid populations: (**A**) yield distribution of the hybrid populations; (**B**) proportion of hybrids in the population exceeding the CK yield.

The phenotypic distribution of the hybrid yield suggests that C229 exhibited strong general combining ability for yield, with earlier DTA and DTS, while PH and EH were shorter in POP2, POP3, and POP5 (Figure 3A and Figure S1). In addition, a comparison of yield with the check indicated that inter-heterotic-group fusion is a viable strategy. There were many hybrids observed that surpassed the CK varieties DH605 and ZD958 in all 15 hybrid populations (Figure 3B). The POP5 population, produced through the aggregation of the X group and the SiPingTou germplasm, when tested with respect to the Improved Reid group, showed the highest number of superior hybrids over the check (Figure 3B). Notably, in the POP5/C229 population, 10.8% of the hybrids exceeded the yield of the high-yielding CK, ND678. Substantial genetic variation was observed from the inter-heterotic-group fusion, allowing for the selection of superior hybrids.

### 2.3. Predicted Accuracy in DH and Hybrid Populations Using Different Models

The predictive accuracies of two machine learning methods and three linear models for traits were compared and analyzed in the inter-heterotic-group DH and test-cross populations. Under the four scenarios, BayesB, RKHS, and RRBLUP outperformed both SVM and RF in predicting PH and EH. The performance of each model varied across the four scenarios for predicting DTA and DTS. Within the DH populations, SVM showed higher accuracy than RRBLUP in predicting DTS, with an accuracy of 0.49. In the DH cross population prediction, SVM achieved the highest accuracy (of 0.21) for both DTA and DTS. Within the hybrid populations, BayesB, RKHS, and RRBLUP outperformed both SVM and RF in predicting DTA and DTS. In the hybrid cross population prediction, RF and RRBLUP exhibited the same predictive accuracy, which was lower than BayesB and RKHS for DTA. However, for DTS, RF demonstrated the same predictive accuracy as RKHS, outperforming RRBLUP and SVM while underperforming BayesB. Overall, BayesB and RKHS—with an accuracy of 0.44—outperformed SVM, RF, and RRBLUP in terms of prediction accuracy across all four scenarios (Table 1). Additionally, the RKHS model is more computationally efficient than BayesB, making it the preferred choice for subsequent analyses.

### 2.4. Prediction Accuracy of DH and Hybrid Within Populations for Different Traits

**Figure 4:**
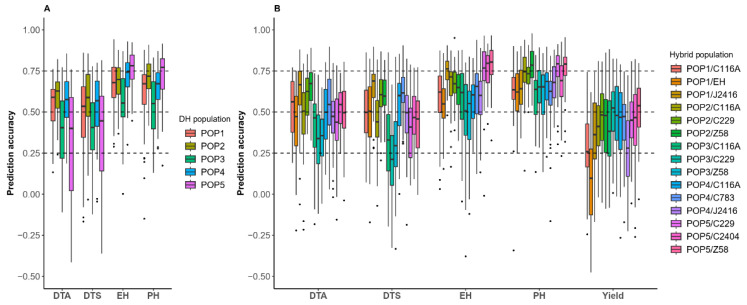
Prediction accuracy of each trait within DH and hybrid populations: (**A**) prediction accuracy of DH populations for different traits; (**B**) prediction accuracy of hybrid populations for different traits. The traits included days to anthesis (DTA), days to silking (DTS), ear height (EH), plant height (PH), and yield.

To evaluate the impacts of populations and traits on the prediction accuracy of inter-heterotic-group DH and test-cross populations, we performed within-population prediction, as illustrated in Figure 4. In the five inter-heterotic-group DH populations, PH and EH exhibit higher prediction accuracy than DTA and DTS, with the prediction accuracies exceeding 0.5 for both PH and EH. POP2 exhibited the highest prediction accuracy for DTA and DTS, reaching 0.63 and 0.59, respectively. In contrast, the prediction accuracies for DTA in POP3 and POP5 were the lowest (at 0.40), while the lowest prediction accuracy for DTS occurred in POP3 (at 0.41). POP5 achieved the highest prediction accuracy for PH and EH, with values of 0.77 and 0.78, respectively. In contrast, the lowest prediction accuracy for POP3 was 0.55 for both PH and EH (Figure 4A). There were also differences in the prediction accuracy of the hybrid populations across different traits. For the trait DTA, the accuracy across the 15 populations ranged from 0.34 to 0.67, with POP2/Z58 and POP1/J2416 exhibiting the highest prediction accuracy. For the DTS prediction, the accuracy ranged from 0.21 to 0.69, with POP1/J2416 showing the highest accuracy. For the EH prediction, the accuracy varied from 0.51 to 0.80, with POP5/Z58 and POP5/C2404 achieving the highest accuracy. For the PH prediction, POP2/Z58 and POP5/C229 had the highest prediction accuracy, ranging from 0.62 to 0.79. Regarding yield, the prediction accuracy within each of the 15 populations ranged from 0.08 to 0.54, with POP5/Z58 exhibiting the highest accuracy among all the hybrid populations (Figure 4B). The prediction accuracy varied across different populations and traits, but all populations achieved prediction accuracies higher than 0.5 for PH and EH.

### 2.5. Prediction Accuracy of Cross DH Populations

**Figure 5:**
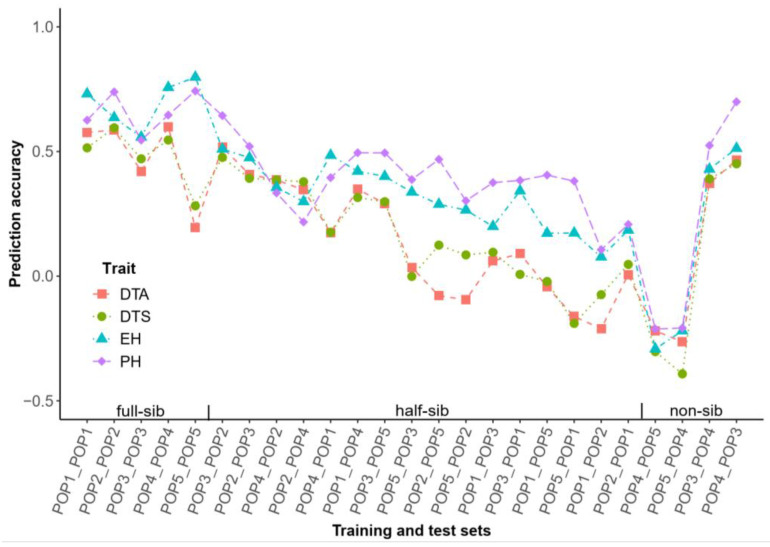
Predictive ability of four traits across the DH populations. The two populations connected with the line represent the training and the test sets, respectively (Training_Test). The four colors in the line graph represent the four traits: days to anthesis (DTA), days to silking (DTS), ear height (EH), and plant height (PH).

Through observing the prediction accuracy for different phenotypes and combinations in the inter-heterotic-group DH populations, we found that the prediction accuracies of PH and EH in the cross DH populations were generally higher than those for DTA and DTS (except for the pairs POP4_POP5, POP2_POP4, POP4_POP2, and POP3_POP2). The average prediction accuracies across all phenotypes for half-sibs were between those of full-sibs and non-sibs, except for POP4_POP2 and POP3_POP2. POP2 and POP3, which belonged to the same heterotic pattern, exhibited the highest mutual prediction accuracy, with average phenotypic prediction accuracies of 0.54 and 0.45, respectively, ranking first and third in terms of the DH cross population prediction accuracy. In the half-sib populations, POP1 and POP2 exhibited the lowest mutual prediction accuracy, while the prediction accuracy between the two unrelated populations—namely, POP5 and POP4—was the lowest. In this experiment, the between-population prediction accuracy of half-sibs varied depending on the different parents. The prediction accuracy for half-sibs of UH306 was higher than that of C229, while C229 outperformed C783. Although an unrelated population relationship existed between POP4 and POP3, both EH and UH306 shared the same heterotic group. Despite this, the average prediction accuracy for all traits in both populations was 0.35. Consequently, the mutual prediction accuracy between POP4 and POP3 was higher than the average when compared to the half-sib predictions involving C783 (Figure 5). The cross population prediction accuracy was more favorable when the no-shared parents of half-sibs belonged to the same heterotic group.

### 2.6. Prediction Accuracy and Classification of Cross Hybrid Populations

**Figure 6:**
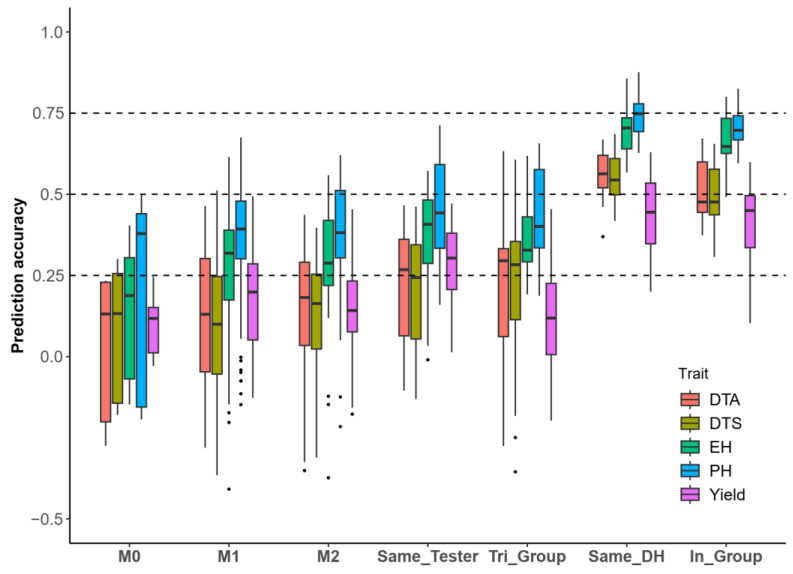
Prediction accuracy of cross hybrid population for different categories: (1) predictions within the populations (In_Group); (2) predictions between different testers within the same DH populations (Same_DH); (3) predictions between different DH populations within the same testers (Same_Tester); (4) predictions within the “triangular” heterotic groups (DH parents and testers in the training and test sets sharing three identical heterotic groups, “Tri_Group”); and (5–7) in addition to the above types, there were 0–2 shared inbred lines between the training and the test sets of the DH parents and testers (M0, M1, and M2, respectively).

We examined the accuracy of cross population predictions for hybrids and found that the radar plot revealed a generally higher prediction accuracy when different testers were combined with the same DH population (Figure S2), and the mean prediction accuracies for DTA, DTS, EH, PH, and yield were 0.56, 0.55, 0.70, 0.74, and 0.43, respectively (Figure 6). In other types of cross population prediction, the POP3/Z58 population exhibited the highest prediction accuracy when predicting POP2/C116A, with prediction accuracies for DTA, DTS, EH, PH, and yield of 0.46, 0.49, 0.56, 0.64, and 0.35, respectively. Additionally, the prediction accuracy between non-common DH populations for some hybrids was also relatively high, particularly in the mutual predictions between POP2- and POP3-matched hybrids. The mean prediction accuracy for POP3-matched hybrids predicting DTA, DTS, EH, PH, and yield for POP2-matched hybrids was 0.37, 0.36, 0.50, 0.62, and 0.34, respectively. Conversely, the prediction accuracy for POP2-matched hybrids predicting POP3-matched hybrids for DTA, DTS, EH, PH, and yield were 0.29, 0.26, 0.46, 0.55, and 0.32, respectively. The second-highest accuracy was observed in the mutual predictions between POP4- and POP2-matched hybrids.

Therefore, we classified the predictions among hybrid populations into seven categories based on the parents of the DH populations and testers, including the following: (1) predictions within the populations (In_Group); (2) predictions between different testers within the same DH populations (Same_DH); (3) predictions between different DH populations within the same testers (Same_Tester); and (4) predictions within the “triangular” heterotic groups (DH parents and testers in the training and test sets sharing three identical heterotic groups “Tri_Group”); and (5–7) in addition to the above types, there were 0–2 shared inbred lines between the training and the test sets of the DH parents and the testers (M0, M1, M2) (Figure 1C). In terms of PH, EH, DTA, and DTS, Figure 6 shows that the prediction accuracy within populations and cross hybrid populations of Same_DH was higher than that of cross population predictions involving Same_Tester or Tri_Group. Furthermore, the accuracy of cross population predictions involving Same_Tester or Tri_Group was generally higher than or similar to those of M0, M1, and M2. In the prediction of yield, the prediction accuracy for Same_DH was higher than that for Same_Tester and better than that for the other types of populations. Overall, the cross population prediction pattern of Same_DH is feasible.

### 2.7. Relationship Between Prediction Accuracy and Genotypic Character of Cross DH or Cross Hybrid Populations

**Figure 7:**
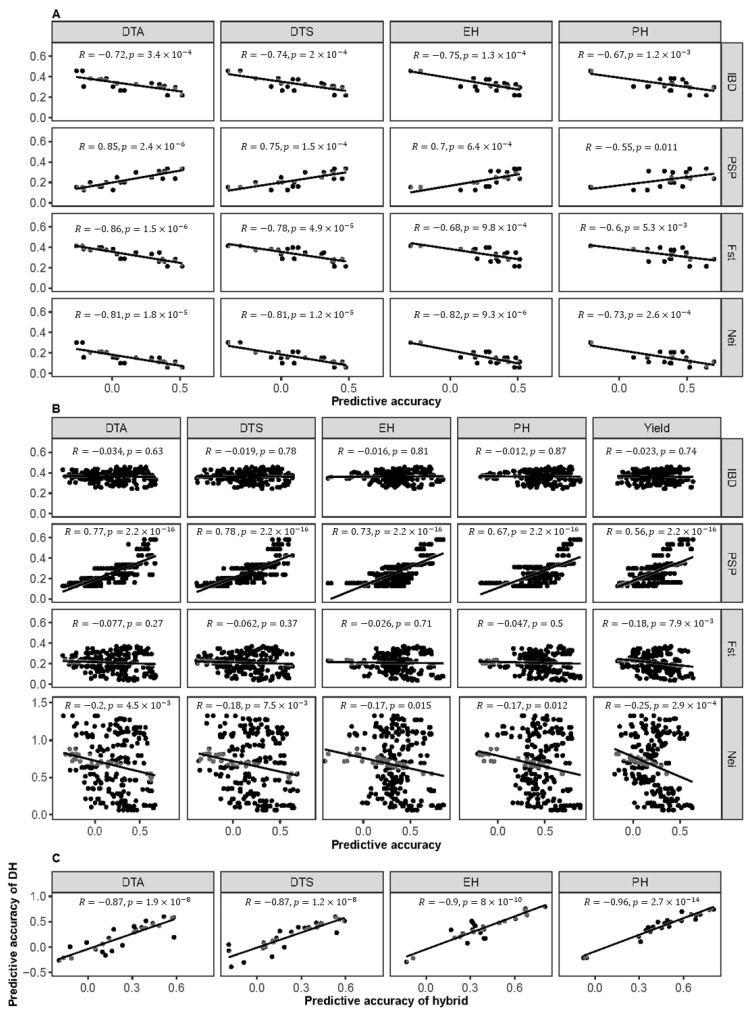
Correlations between cross population prediction accuracy and genotypic character of cross population regarding DH or hybrid population: (**A**) correlations between predictive accuracy and genotypic character of cross DH populations; (**B**) correlations between predictive accuracy and genotypic character of cross hybrid populations; (**C**) correlations between DH and hybrid populations in terms of prediction accuracy.

To investigate whether the genotypic relationship is associated with the cross population prediction, we examined the genotypic indices between populations and found a strong correlation between the prediction accuracy of cross DH populations and the Nei, IBS, Fst, and PSP (the proportion of shared polymorphisms of SNPs between the training and the test sets) indices, with absolute correlation coefficients ranging from 0.55 to 0.86, all of which were statistically significant (Figure 7A). The prediction accuracy for the cross hybrid populations was strongly correlated only with the PSP index, with correlation coefficients ranging from 0.56 to 0.78 (Figure 7B). Regarding the prediction accuracy for the cross DH populations, there was a single correlation coefficient while, for the hybrid populations, there were nine distinct correlation coefficients corresponding to each DH population. We first calculated the mean prediction accuracy across the nine hybrid populations and then assessed the correlations in the prediction accuracy between the DH populations and their corresponding hybrid populations. The results showed a strong correlation between the prediction accuracy of the DH populations and their corresponding hybrid populations for different phenotypes, with correlation coefficients ranging from 0.87 to 0.96, all of which were highly significant (Figure 7C). The cross population prediction accuracy was correlated with cross population genotypic relationships, and a significant correlation was observed between the DH and the hybrid populations.

## 3. Discussion

The creation of inter-heterotic-group DH populations is an important method for heterotic group innovation. The integration of DH and genomic selection (GS) techniques in these populations can significantly accelerate the selection process. In this study, we utilized elite inbred lines from four heterotic groups to generate a connected segregating population through inter-heterotic-group line crossing (CSPIC). We then examined whether the improvement of inter-heterotic-group DH lines was effective, as well as the prediction accuracy both within- and between- DH and hybrid populations.

### 3.1. Superior Hybrids Can Be Produced Through Inter-Heterotic-Group Crossing

Elite line improvement typically involves selfing progeny from a cross between two inbreds coming from same heterotic group, that is, intra-heterotic-group crossing. These inbreds are subsequently evaluated for their yield performance when test-crossed with an inbred from a different heterotic group [43,45]. However, through inter-heterotic-group crossing—a potential method for the creation of new heterotic groups—we used five elite lines from four heterotic groups to create five new inter-heterotic-group populations. Those DH populations exhibited distinct super-parental phenotypes, indicating that inter-heterotic-group germplasm fusion can generate abundant genetic variation. For example, the DTS phenotype of POP5 ranged from 60.6 to 74.6 days, while both parents had a DTS phenotype of 68 days. In addition, Bernardo has shown that the breeding of inbreds between heterotic groups depended mainly on the choice of testers [43]. This breeding practice demonstrates the existence of the triangular heterotic pattern and highlights the potential for hybridization between specific pairings [44]. Using the triangular heterotic model for inter-heterotic-group improvement and heterotic group testing, we confirmed its effectiveness in practice. Moreover, this approach provides a definite heterotic pattern for inter-heterotic-group improvement, and our experimental results further support the feasibility of inter-heterotic-group crossing. The proportion of those surpassing the CK hybrid (ZD958) in the hybrid populations ranged from 1.1% to 53%, and 10.8% of the hybrids in the population POP5/C229 surpassed the high-yielding hybrid ND678 (CK), indicating that producing DH line test-cross hybrids through an inter-heterotic-group strategy is a viable option (Figure 3B). Other studies have also indicated that inter-heterotic-group crossing for the selection of lines is feasible. A prominent example of this is Mo17 in the United States, which contains 50% Lancaster and 50% Krug (Reid) [40]. In China, a large number of maize hybrids and commercial maize varieties have been introduced from abroad, leading to the selection of numerous maize inbred lines such as Qi319, Jing724, and so on, as well as new heterotic groups, such as the P and X groups, among others, originating from these hybrid varieties. Germplasm improvement through inter-heterotic-group crossing should be the major path forward in future maize breeding.

### 3.2. RKHS Model Exploring the Best Prediction Accuracy Among the GS Models

In this study, we primarily applied five models, including two machine learning methods: RF and SVM. Among these models, BayesB, RKHS, and RRBLUP generally outperformed RF and SVM in terms of prediction accuracy, with values of 0.44, 0.44, and 0.43, respectively, suggesting that machine learning models exhibit lower prediction accuracy for different maize populations. Similarly, some studies have shown that SVM has a poor prediction effect in the hybrid rice NCII population [46]. Heslot et al. have demonstrated the application of multiple models across various crops, showing that RKHS generally outperforms other models in terms of accuracy, while support vector machines exhibited poor performance on these datasets [27]. Both the RKHS and BayesB models exhibited the highest average prediction accuracy of 0.44 in our study; however, they did not consistently provide the best predictions across all scenarios and traits. This is in line with the findings of Azodi et al., who noted that no single model excels in predicting all traits [47]. Therefore, the choice of model should be based on the specific context. To streamline subsequent comparisons, this study selected RKHS for further analysis, as it offered the best overall prediction accuracy and faster computation.

Traits are an important factor influencing the accuracy of GS predictions, with the variation in prediction accuracy primarily reflecting differences in heritability. Daetwyler et al. have demonstrated that traits with a higher heritability tend to yield higher predictive accuracy [48]. The results of our research were consistent with previous studies, showing that the prediction accuracy for PH and EH is generally higher than that for DTA, DTS, and yield. However, some studies have reported exceptions to this trend [17,49,50]; for example, Heffner et al. found that the heritability of grain softness in the parental population Cayuga × Caledonia was 0.88, while the prediction accuracy was 0.37. In our study, a similar result was observed in the hybrid populations, where the prediction accuracy for PH in POP1/J2416 was lower compared to DTS. In the DH population, the prediction accuracy for DTA and DTS in POP5 was also low, likely due to the small phenotypic differences between the parental lines in this population. The accuracy of yield prediction for POP1/EH was the lowest at 0.06, which may be attributed to the fact that the tester for the EH inbred line originated from northeast China, while the phenotypic data were collected from the Huanghuai Plain. This environmental variation likely reduced the genetic variation in the POP1/EH population, leading to a lower prediction accuracy.

### 3.3. Cross Population Prediction in Half-Sibs with Non-Shared Parents from the Same Heterotic Group Exhibiting Potential Accuracy in DH Population

In the prediction between different DH populations, a clear trend emerged: the prediction accuracy for full-sib populations was better than for half-sib populations (Figure 5). Moreover, the prediction accuracy for two DH populations without a genetic relationship was negative, which aligns with previous studies [51,52,53]. The negative prediction result for the non-sib populations was likely due to differences in the estimated SNP effects between the two populations [54], indicating that predictions between completely unrelated populations hold limited practical value in breeding. However, when the non-shared parent of the half-sib came from the same heterotic group, the prediction results were much more reliable, ranging from 0.34 to 0.64 (Figure 5). This suggests that the accuracy of predictions is largely influenced by the pedigree relationship, which is consistent with the results from the PCA analysis [51,52,55] (Figure 2A). Although POP4 and POP3 were unrelated populations, they showed high accuracy in mutual prediction, possibly as their genetic structures were similar and both populations shared a parent from the same heterotic group.

### 3.4. Cross Population Prediction Among Hybrids from the Same DH Population Expressing Prominent Accuracy

In terms of the prediction accuracy obtained for the hybrid cross populations, we observed that the accuracy of Same_DH was significantly higher than that of the other cross population predictions. This finding supports a potential pattern for the cross population prediction of hybrid phenotypes. When testing multiple lines within the same DH population, we can generate a training set using one tester and predict the performance of populations with the other testers. This approach helps to reduce the cost of field experiments. The prediction accuracies for Same_DH were 0.60, 0.60, 0.76, 0.82, and 0.49, with these being higher than those of In_group (with values of 0.51, 0.49, 0.63, 0.67, and 0.41), for DTA, DTS, EH, PH, and yield, respectively. This difference may be due to the sampling methods, as the DH lines in the training and the test sets for In_group predictions were distinct, whereas Same_DH may involve some overlap of the DH lines between the training and the test sets. Consequently, we categorized the cross population prediction types of Same_DH into three distinct categories and compared them with In_group. We found that the prediction accuracy for Same_DH was higher than that of rand_DH, and rand_DH, in turn, outperformed diff_DH. The results support the validity of our hypothesis (Figure S3B). What this means is that the highest prediction accuracy for cross population predictions of the same DH hybrids was achieved when the DH line from the test set hybrid was included in the training set. Additionally, when the testers came from different heterotic groups, the cross population predictions remained effective, particularly for traits such as DTA, DTS, EH, and PH (Figure S3A). The Same_DH model proved to be a viable approach for cross population prediction in hybrid populations, yielding prediction accuracies comparable to those of within-population predictions.

### 3.5. The Proportion of Shared Polymorphisms (Considering SNPs) Between the Training and the Test Sets (PSP) Correlates with the Cross Population Prediction of Both the DH and Hybrid Populations

As the predictive accuracy of GS is closely linked to the genomic similarity between the training and the test populations [56], evaluating the genetic relationships between populations is crucial. We analyzed the correlations between the prediction accuracy and various genetic indices, including Nei, IBD, Fst, and PSP, in both the DH and the hybrid populations. The results revealed that the cross population prediction in both the DH and the hybrid populations was significantly influenced by the PSP index, with the coefficient of variation ranging from 0.55 to 0.86. This suggests that the PSP index can be used to assess the reliability of cross population predictions. Furthermore, we observed a high correlation between the prediction accuracy in the DH populations and the corresponding hybrid populations, with the coefficient of variation ranging from 0.87 to 0.96. This is consistent with the predicted results. For the DH cross population predictions, the highest mutual prediction accuracy was observed between POP2 and POP3 in the half-sib population, followed by the second-highest accuracy between POP2 and POP4. Similarly, for the hybrid cross population predictions, the highest prediction accuracy was observed in the hybrids derived from POP2- and POP3-matched populations, with the second-highest accuracy found in the mutual predictions between POP2- and POP4-matched hybrids. Specifically, when the prediction accuracy was high in the DH populations, it could be effectively extended to their respective hybrids.

## 4. Materials and Methods

### 4.1. Plant Materials, Experimental Design, and Phenotypic Data Collection

In this experiment, four inbred lines (C783, C229, C116A, and EH) bred by the laboratory and one introduced line (UH306) were used as the base materials for the production of the DH populations. These five parent lines belonged to four divergent heterotic groups: X group (C783), Improved Reid group (C229), SiPingTou germplasm (C116A), and Early-maturity germplasm (UH306, EH). Five DH populations were constructed using these five inbred lines, and the five DH populations were C783 × C229, C783 × UH306, C783 × EH, C229 × UH306, and C783 × C116A, which were labeled as POP1–5, respectively. Then, the five DH populations were tested against three inbred lines with heterosis, which were from the SiPingTou germplasm (C116A, J2416), Improved Reid group (C229, C2404, Z58), X group (C783), and Early-maturity germplasm (EH), yielding a total of 15 hybrid populations. The specific hybridization scheme is shown in Table S1.

The DH lines were planted in Beijing (N40°8′ E116°11′) in 2020, Xinxiang (N35°9′ E113°47′) and Hebi (N35°40′ E114°18′) in 2021, and Sanya (N18°23′ E109°11′) in 2021 and 2023. We used the augmented alpha design, setting every 20 accessions as a block. Each block consisted of nineteen rows of accessions and one constant accession of C116A, planted in random order. In the five environments, phenotyping was recorded for two agronomic traits: plant height (PH, cm) and ear height (EH, cm). Days to anthesis (DTA, days) and days to silking (DTS, days) were recorded in three environments: Beijing in 2020, Xinxiang in 2021, and Sanya in 2021. The number of DH phenotypes collected in POP1–5 was 124, 97, 134, 91, and 89, respectively (Table S1).

Hybrids were planted in Handan (N36°21′ E114°44′), Xinxiang (N35°9′ E113°47′), Hebi (N35°40′ E114°18′), Jining (N34°58′ E116°13′), and Jinan (N37°24′ E117°14) from 2019 to 2021. We used the augmented alpha design, and each block consisted of twenty-one accessions and three constant accessions of DengHai605 (DH605), ZhongNongDa678 (ND678), and ZhengDan958 (ZD958), planted in random order. The data on maize yield were collected across 15 different environments, but PH and EH were collected in 12 environments, excluding Handan in 2020 and 2021 and Jinan in 2020. For DTA and DTS, we investigated nine environments in Hebi and Xinxiang from 2019 to 2021, Handan and Jining in 2019, and Jining in 2020. We collected field phenotypic data from 1568 hybrids. In the field, each accession was planted in a one-row plot for the DHs and a two-row plot for the hybrids, with 0.6 m of row space and 0.25 m of plant space.

### 4.2. Phenotypic Data Analysis and Heritability Estimation

The raw phenotypic data were analyzed using a linear mixed model via the lme4 package in R (V4.1.2) [57]. Best linear unbiased estimation (BLUE) was used to calculate the DH lines and the hybrids. In the model, the following equation was used:

$$y_{ijk}=\mu +g_{i}+e_{j}+b_{k}\left(e_{j}\right)+\varepsilon ,$$

where $y_{ijk}$ is the mean phenotypic value of the ith DH line or hybrid in the kth block of the jth environment, μ is the overall mean, $g_{i}$ is the genotype effect of the ith DH or hybrid (fixed effects), $e_{j}$ is the effect of the jth environment (random effects), $b_{k}(e_{j})$ is the effect of the kth block nested within the jth environment (random effects), and ε is the random error. The heritability was calculated using variance components estimated from the above model. The following equation was used to estimate the heritability on a plot-mean basis:

$$H^{2}=\frac{V_{g}}{V_{g}+\frac{V_{e}}{l}},$$

where $V_{g}$ is the genotypic variance component, $V_{e}$ is the error variance, and l is the number of environments.

### 4.3. Genotyping and Genotypic Data Analysis

Paired-end 150 sequencing was performed on the DH lines, the testers, and the parents of the DH populations using the BGI DNBSEQ-T7 platform (BGI, Shenzhen, Guangdong Province, China). The average number of raw reads for the DH lines and the testers was 42,248,045, and the average number of reads for the parents of the DH populations was 186,780,554. Quality control of the raw reads was performed using Fastp (V0.20.0) with the default parameters [58]. The filtered sequencing data were aligned to the reference genome (B73_RefGen_v5) using BWA (V0.7.17), followed by sorting, indexing, alignment, and statistics on alignment results using Samtools (V1.10) [59,60]. The genome coverage of the DH lines and the testers was 0.76, with an average sequencing depth of 3.6×. For the parents of the DH populations, the genome coverage was 0.86, with an average sequencing depth of 12.9×. GATK (V4.2.0) was used to detect and extract segregating SNPs, followed by basic parameter filtering with the following filtering criteria: “QUAL < 30.0||FS > 60.0||QD < 2.0||SOR > 3.0||MQ < 40.0||MQRankSum < −12.5||ReadPosRankSum < −8.0” [61]. After filtering SNPs, vcftools (V0.1.16) was used again for filtering, with a minimum sequencing depth of 1× and a minimum quality of Q30 [62]. Unaligned segments were removed, resulting in a final filtered SNP count of 300,521,103.

Plink was used to separately filter the five DH populations, with the filtering parameters set as follows: (a) less than 40% missing values of SNPs; (b) less than 30% missing values of individuals; and (c) a heterozygosity rate of individuals less than 10% [63]. Then, the filtered data were imputed using BEAGLE version 5.0 [64] and, for each DH population, a chi-square test at a significance level of 0.001 was used for filtering the 1:1 segregation. After filtering, the remaining number of SNPs in POP1–POP5 were 210,056, 181,743, 74,351, 130,781, and 136,785, respectively. The genotypes of the five populations were then merged, with the merging conditions set to retain SNPs shared by all five populations and other loci with variation in at least one population. The final total number of SNP markers in the merged population was 108,898.

The genotypes of hybrids were obtained with the cleaned SNPs (N = 108,898) of DHs and testers using TASSEL V5.2 [65]. When predicting within a single population or between two populations, we extracted the genotypes of the populations and filtered out the non-segregating SNPs. Principal component analysis (PCA) was used to assess the level of genetic structure using the Plink software (V1.9) [63]. To examine the correlation between the genotyping affinity of cross populations and the prediction accuracy, four indices were used to evaluate the genotyping affinity of cross populations: (a) the proportion of shared polymorphisms between the training and the test sets relative to the SNPs shared by the five populations (PSP); (b) Nei’s genetic distances between the training and the test sets, calculated using the R package “adegenet” (Nei)(V2.1.11) [66]; (c) the IBS index, which is the average distance matrix between the training and the test sets, calculated using the TASSEL V5.2 software (IBS) [65]; and (d) the Fst index, calculated using vcftools (V0.1.16), with the single point calculation method (Fst) [62].

### 4.4. Genomic Prediction

GP was performed for the hybrid and DH populations using five models: ridge regression best linear unbiased prediction (RRBLUP) [21], BayesB [2], Reproducing Kernel Hilbert Space (RKHS) [31,32], support vector machine (SVM) [67,68], and random forest (RF) [69]. The RRBLUP method, which is based on a restricted maximum likelihood (REML) approach, was implemented using the R package “rrBLUP”(V4.6.3) [22]. Additionally, we applied the scaled-t mixture model (BayesB), which uses a mixture distribution that includes a point mass at zero and a univariate scaled t-distribution to fit the models, implemented using the R package “BGLR” (V1.1.3) [70]. The basic model is as follows:

$$y=1_{n}\mu +Z\alpha +\varepsilon ,$$

where y is the vector of phenotypes, $1_{n}$ is the n-dimensional vector of ones, μ is the overall mean, α is a vector of random regression coefficients of all the marker effects, Z is a genotypic matrix for markers, and ε is a vector of residuals. The alternative methods discussed here primarily differ in terms of the specific priors used for α. For RRBLUP, α~N (0, I$\sigma_{\alpha }^{2}$), and $\sigma_{a}^{2}$ has a scaled inverse chi-square distribution. Meanwhile, BayesB employs a mixture distribution that includes a point of mass at zero and a univariate scaled t-distribution.

RKHS is a semi-parametric regression model that was first applied to marker genotypes by Gianola et al. [71]. The RKHS model was implemented using the R package “BGLR”(V1.1.3). The model is calculated as follows:

$$y=1_{n}\mu +k_{h}\alpha +\varepsilon$$

where y is the vector of phenotypes; $1_{n}$ is the n-dimensional vector of ones; μ is the overall mean; $k_{h}$ is a kernel function that maps the input data to a higher-dimensional space, such that the data can be more easily separated; α is assumed to have an independent prior distribution α~N (0, $k_{h}\sigma_{\alpha }^{2}$); and ε is a vector of residuals.

RF is an ensemble machine learning method composed of multiple decision trees [69]. These decision trees are generated using a randomized tree-building algorithm, which creates several trees by randomly sampling the original training set, allowing for certain items to appear more than once. When RF is used for regression, the final output is the average of the predictions from all decision trees [72]. We implemented this method using the R package “randomForest”(V4.7-1.2) [73].

SVM is a non-parametric algorithm proposed by Cortes and Vapnik [68]. It was first applied for classification; however, at present, it is widely used for both regression and classification. We implemented SVM using the R package “e1071”(V1.7-16) [74].

### 4.5. Calculation of Prediction Accuracy

For within-population predictions, 77 DH or hybrid accessions were randomly, and a five-fold cross-validation approach was employed, which was repeated 10 times in order to evaluate the accuracy of the GS models. For the cross population predictions, to maintain consistency with the within-population predictions, 55 accessions were sampled from the training set and 12 accessions from the test set. This procedure was repeated 50 times to evaluate the prediction accuracy. The prediction accuracy for each sample was calculated according to the Pearson correlation between the input trait values and the genomic estimated breeding values (GEBVs) from the given GS model. We selected the median Pearson correlation coefficient from the 50 repetitions as the representative measure of prediction accuracy.

### 4.6. DH Population Prediction Classification

We divided the DH populations into three categories based on the presence of common DH parents between the DH training and test sets as follows: (I) no common parents between the training and the test sets (non-sib); (II) half-sibling populations (half-sib); and (III) predictions within the populations (full-sib). Prediction type II was further divided into three categories as follows: half-sibling populations with the shared parental lines (C783, C229, UH306), and half-sib of C783 with non-shared parents from the same heterotic group or different heterotic groups. For the specific classification of DH populations prediction types, refer to Table S2.

### 4.7. Hybrid Population Prediction Type Division

Each hybrid population primarily consisted of three components: two founder lines of the DH populations and a tester. All hybrid populations in this study were derived from the “triangle” heterotic models, which refers to the production of hybrids through a single cross using inbred lines selected from two of the three heterotic groups [44]. The DH founder lines and the testers consisted of four heterotic groups (Improved Reid group, SiPingTou germplasm, Early-maturity germplasm, X group; see Figure 1). Based on the difference between the training and the test sets in the two DH population parents and the tester, the prediction types were divided into seven categories: (1) In_Group: the training and the test sets were derived from the same hybrid population; (2) Same_DH: the two DH population parents remained unchanged, but the training and the test sets used different testers; (3) Same_Tester: the training and test sets used the same tester, but there was at least one difference in the DH founder liners of the two populations; (4) Tri_Group: DH parents and testers in the training and test sets shared three identical heterotic groups. For example, the mutual prediction between POP1/C116A and POP5/C229 fell under the Tri_Group type; and (5–7) M0, M1, and M2: the training and the test sets had 0–2 consistent materials in terms of the parents of the DH populations and the testers, but they did not have the same DH populations and the same testers (Figure 1C). For the specific classification of hybrid population prediction types, refer to Table S3.

## 5. Conclusions

This study revealed the following several key findings: (a) The improvement in DH lines through inter-heterotic-group line crossing is feasible, and this method can effectively be used to select superior hybrids. (b) In the DH cross population prediction, the prediction accuracy of half-sibs was higher than that of non-sibs, and when the non-shared parent of the half-sib population belonged to the same heterotic group, the cross population prediction accuracy was further increased. (c) In the cross hybrid population prediction, the prediction accuracy for hybrids that shared the same DH population was the highest. (d) The PSP index for cross populations showed a high correlation with the prediction accuracy in both DH and hybrid populations. (e) A strong correlation was observed between the prediction accuracy of the DH and hybrid populations in the cross population prediction.

## Figures and Tables

**Table 1:** Mean prediction accuracy for each model across different traits in four scenarios.

Prediction Scenario	Trait	RKHS	BayesB	RRBLUP	SVM	RF
Prediction within DH populations	DTA	0.52	0.54	0.51	0.49	0.44
	DTS	0.51	0.49	0.47	0.49	0.44
	EH	0.69	0.69	0.69	0.59	0.56
	PH	0.68	0.68	0.67	0.57	0.56
	Average	0.60	0.60	0.58	0.53	0.50
Prediction within hybrid populations	DTA	0.51	0.51	0.50	0.47	0.48
	DTS	0.48	0.48	0.48	0.45	0.47
	EH	0.66	0.66	0.65	0.56	0.56
	PH	0.69	0.69	0.70	0.56	0.58
	Yield	0.41	0.41	0.39	0.35	0.36
	Average	0.56	0.56	0.56	0.48	0.49
Cross population prediction of DH	DTA	0.19	0.19	0.19	0.21	0.18
	DTS	0.20	0.20	0.20	0.21	0.20
	EH	0.36	0.36	0.36	0.29	0.28
	PH	0.41	0.39	0.41	0.35	0.33
	Average	0.29	0.28	0.29	0.27	0.25
Cross population prediction of hybrid	DTA	0.23	0.23	0.22	0.21	0.22
	DTS	0.21	0.22	0.20	0.20	0.21
	EH	0.37	0.37	0.36	0.31	0.32
	PH	0.45	0.43	0.45	0.38	0.39
	Yield	0.23	0.24	0.21	0.22	0.22
	Average	0.32	0.31	0.31	0.28	0.28
Overall mean		0.44	0.44	0.43	0.39	0.38

## Data Availability

All relevant data are within the paper and its Supplementary Materials.

## Supplementary Materials

The following supporting information can be downloaded at: https://www.mdpi.com/article/10.3390/ijms26062662/s1.
