# Supplementary material for: Genomic Prediction for Germplasm Improvement Through Inter-Heterotic-Group Line Crossing in Maize
Source: Int J Mol Sci. 2025 Mar 15;26(6):2662. doi: 10.3390/ijms26062662 (PMC11942448; doi:10.3390/ijms26062662)
Supplement: Supplementary file 1 [file ijms-26-02662-s001.zip › Figure.S1.Phenotypic distribution of the DH and the hybrid populations.pdf]

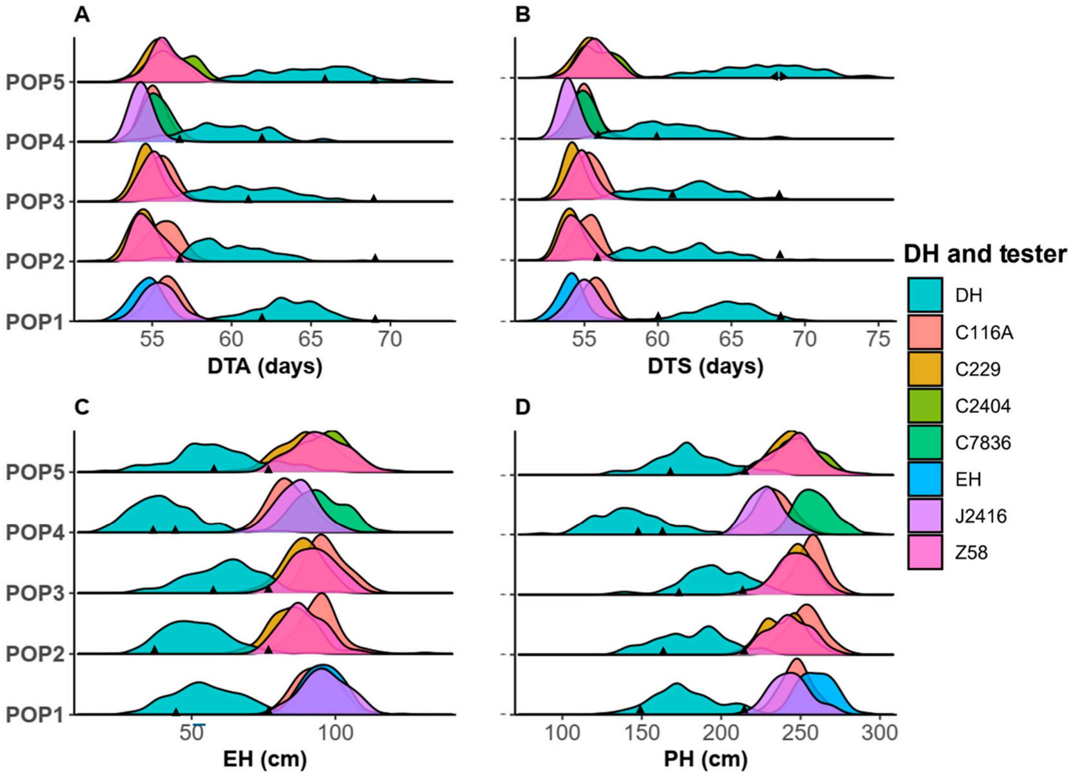

Figure S1. Phenotypic distribution of the DH and the hybrid populations. (A) DTA, days to anthesis; (B) DTS, days to silking; (C) EH, ear height; (D) PH, plant height. The black triangle on the X-axis represents the phenotypes of the parents of the DH population.
