# Supplementary material for: Genomic Prediction for Germplasm Improvement Through Inter-Heterotic-Group Line Crossing in Maize
Source: Int J Mol Sci. 2025 Mar 15;26(6):2662. doi: 10.3390/ijms26062662 (PMC11942448; doi:10.3390/ijms26062662)
Supplement: Supplementary file 1 [file ijms-26-02662-s001.zip › Figure.S2.Prediction accuracy of cross hybrid populations for different phenotypes.pdf]

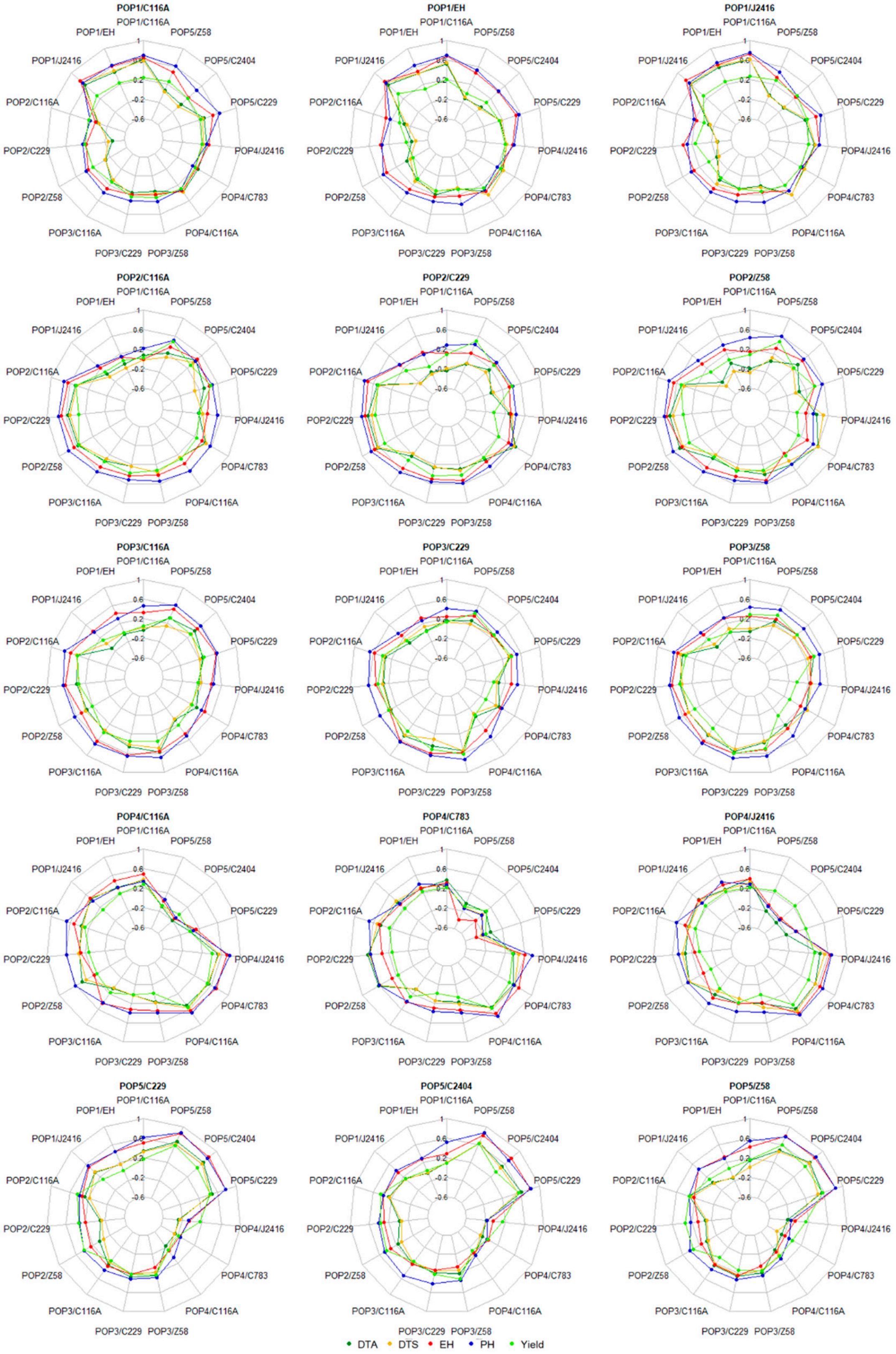

Figure S2. Prediction accuracy of cross hybrid populations for different phenotypes. The bold subheadings above each plot represent the training set, while the labels on the spider plots indicate the corresponding test sets. The traits include days to anthesis (DTA), days to silking (DTS), ear height (EH), plant height (PH), and yield, each represented by a different color.
