# Supplementary material for: Genomic Prediction for Germplasm Improvement Through Inter-Heterotic-Group Line Crossing in Maize
Source: Int J Mol Sci. 2025 Mar 15;26(6):2662. doi: 10.3390/ijms26062662 (PMC11942448; doi:10.3390/ijms26062662)
Supplement: Supplementary file 1 [file ijms-26-02662-s001.zip › Figure.S3.The impacts of different tester sources and training set samplings on the prediction of hybrid populations within the same DH population.pdf]

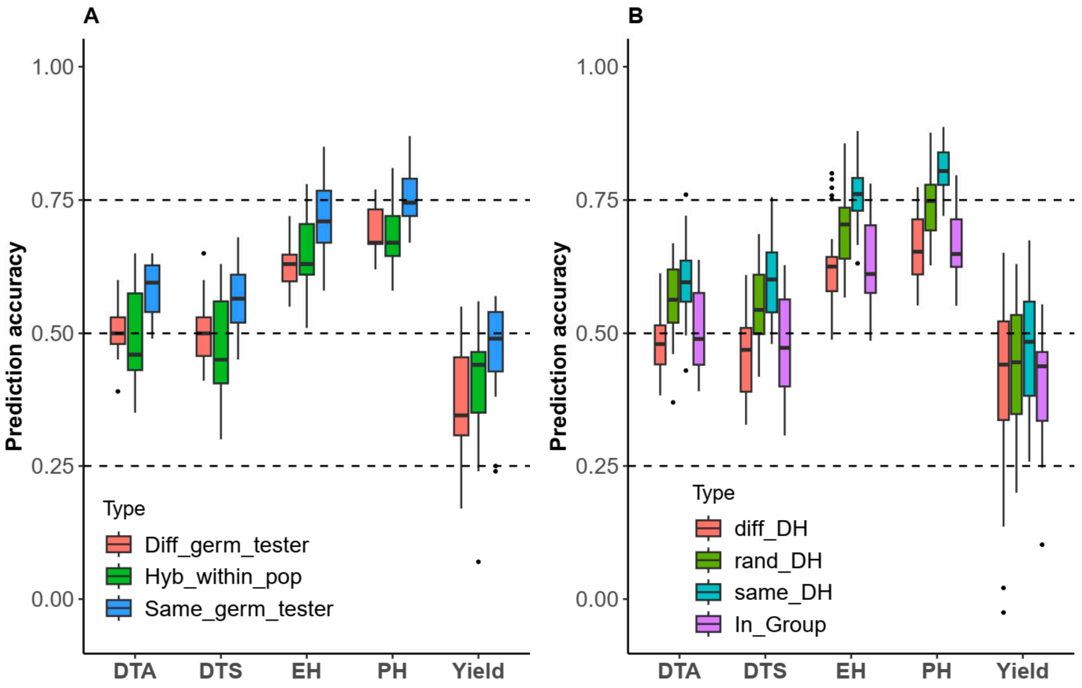

Figure S3. The impacts of different tester sources and training set samplings on the prediction of hybrid populations within the same DH population. (A) In cross population prediction of hybrids within the same DH populations, the testers in the training and the test sets can be classified into the same or different germplasm groups (Diff_germ_tester or Same_germ_tester), and prediction accuracy within hybrid populations (Hyb_within_pop); (B) the sampling methods for the training set were categorized into three types: 1) The training and the test sets have no common DH lines (diff_DH); 2) the training and the test sets are randomly sampled (rand_DH); 3) the training set includes the DH lines of the test set (same_DH). In_Group is consistent with the definition mentioned above (Predictions within the hybrid population).
